# Supplementary material for: Dilution of specialist pathogens drives productivity benefits from diversity in plant mixtures
Source: Nat Commun. 2023 Dec 18;14:8417. doi: 10.1038/s41467-023-44253-4 (PMC10728191; doi:10.1038/s41467-023-44253-4)
Supplement: Supplementary file 1 — Supplementary Information [file 41467_2023_44253_MOESM1_ESM.pdf]

Supplementary Information

Dilution of specialist pathogens drives productivity benefits from diversity in plant mixtures

Guangzhou Wang[1,2*], Haley M. Burrill[2,3,4], Laura Y. Podzikowski[2,3], Maarten B. Eppinga[5], Fusuo Zhang[1], Junling Zhang[1], Peggy Schultz[2,6] and James D. Bever[2,3*]

[1]State Key Laboratory of Nutrient Use and Management (SKL-NUM), College of Resources and Environmental Sciences, National Academy of Agriculture Green Development, China Agricultural University, Beijing 100193, People's Republic of China

[2]Kansas Biological Survey, University of Kansas, Lawrence, Kansas 66045, USA

[3]Department of Ecology and Evolutionary Biology, University of Kansas, Lawrence, Kansas 66045, USA

[4]The Institute of Ecology and Evolution, University of Oregon, Eugene, OR 97403, USA

[5]Department of Geography, University of Zurich, Winterthurerstrasse 190, 8057 Zürich, Switzerland

[6]Environmental Studies Program, University of Kansas, Lawrence, Kansas 66045, USA

***Correspondence:**

Dr. Guangzhou Wang, E-mail: wanggz@cau.edu.cn

Dr. James D. Bever, E-mail: jbever@ku.edu

This file includes:

Supplementary Figures 1-12 (Pages 3-17)

Supplementary Tables 1-8 (Pages 18-25)

Supplementary Discussion (Pages 26-29)

Supplementary Methods (Pages 30-33)

Supplementary References (Pages 34)

Supplementary Figures

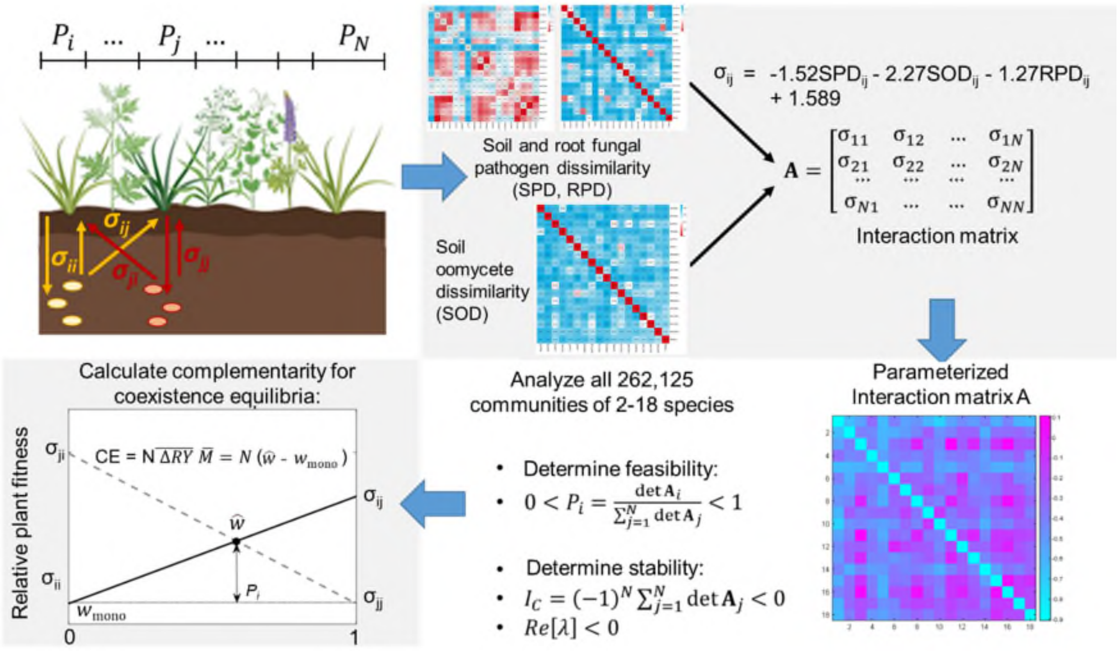

Supplementary Fig. 1 | Schematic overview of how the empirical data was used in a theoretical modelling approach. The general feedback model utilized considers that host plants' modification of the environment affects their own fitness (i.e. conspecific effects) as well as the fitness of other plants (heterospecific effects). This study's empirical relationships linking pathogen dissimilarity to soil-community mediated interactions were used to parameterize these conspecific and heterospecific effects, yielding an interaction matrix determining community dynamics. Community dynamics were inferred from the feasibility of the coexistence equilibrium point, the real part of the leading eigenvalue of the Jacobian matrix evaluated at this point (Re[λ]) and community-level feedback (I_C, see Supplementary Information for details). Subsequently, for plant communities that were feasible and stable, the strength of the complementarity effect could be calculated, and its relationship with predicted plant-soil feedback effects and pathogen dilution be inferred (see Methods for details). Figure partly created with BioRender.com.

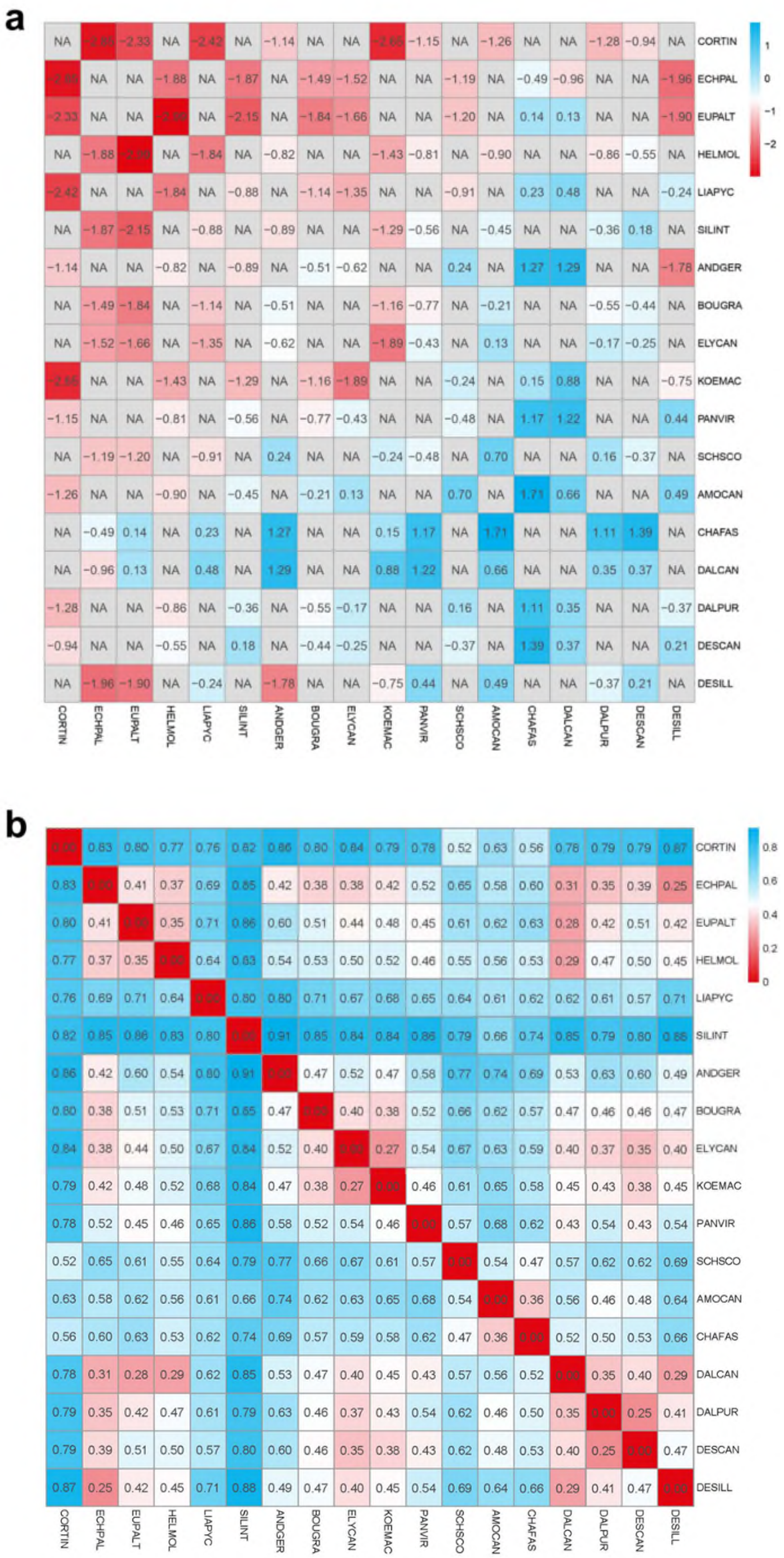
a
CORTIN
ECHPAL
EUPALT
HELMOL
LIAPYC
SILINT
ANDGER
BOUGRA
ELYCAN
KOEMAC
PANVIR
SCHSCO
AMOCAN
CHAFAS
DALCAN
DALPUR
DESCAN
DESILL
1
0
−1
−2
b
0.8
0.6
0.4
0.2
0

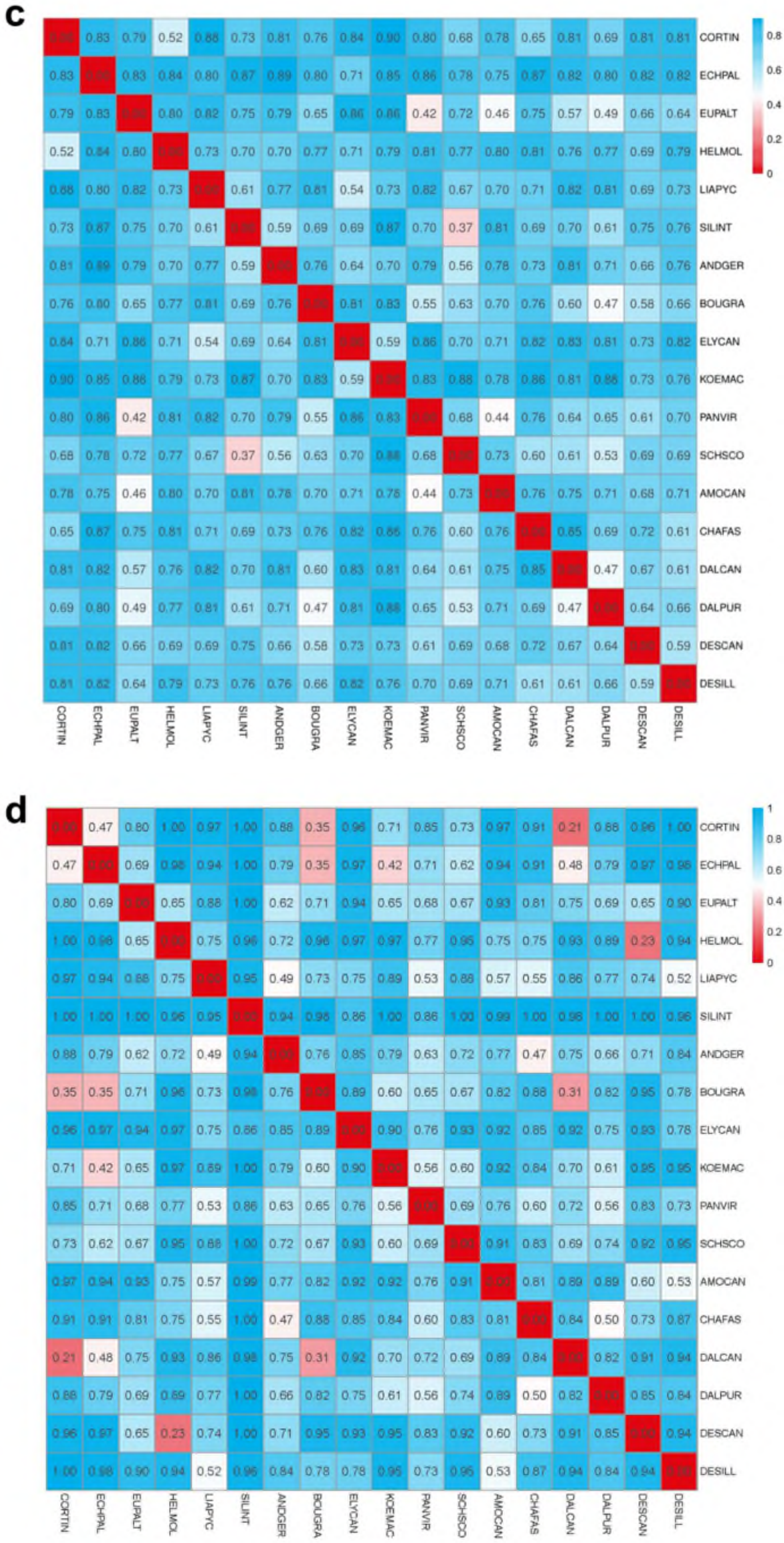

Supplementary Fig. 2 | Pairwise feedbacks, soil and root pathogen dissimilarities

between individual species. a-c Measured pairwise feedback effects in greenhouse assays **(a)**, soil fungal pathogen dissimilarity **(b)**, soil oomycete dissimilarity **(c)** and root fungal pathogen dissimilarity **(d)** between individual species. Source data are provided as a Source Data file.

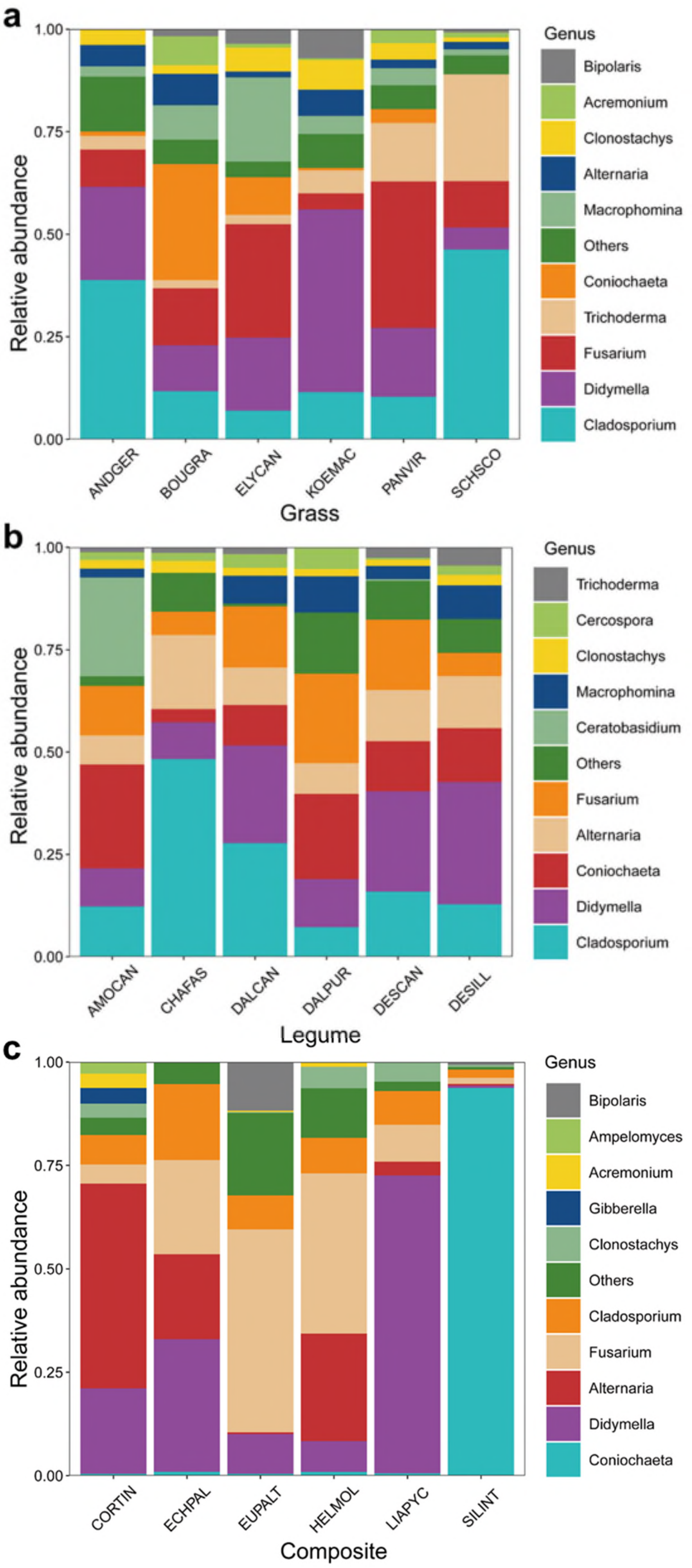

Supplementary Fig. 3 │ The relative abundance of soil fungal pathogens. a-c The relative abundance of soil fungal pathogens at the genus level for each plant species of grass **(a)**, legume **(b)** and composite **(c)** groups. Source data are provided as a Source

Data file.

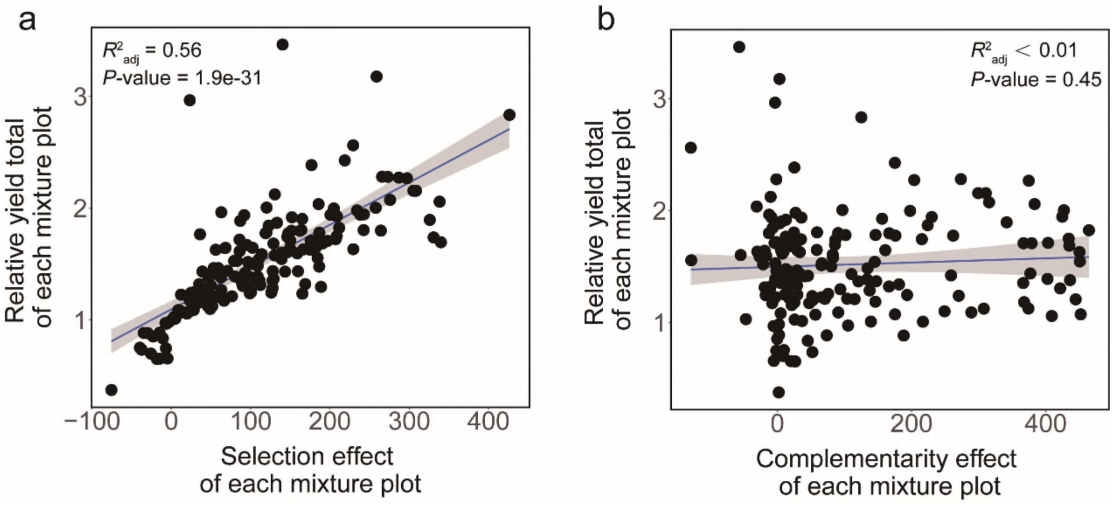

Supplementary Fig. 4 | The regressions between relative yield total and biodiversity effects. a,b, relationships between complementarity effect **(a)**, selection effect **(b)** and relative yield total of field mixture plots. The solid blue line indicates the fitted relationship and the lack gray background indicates the 95% confidence interval. N = 168. Source data are provided as a Source Data file.

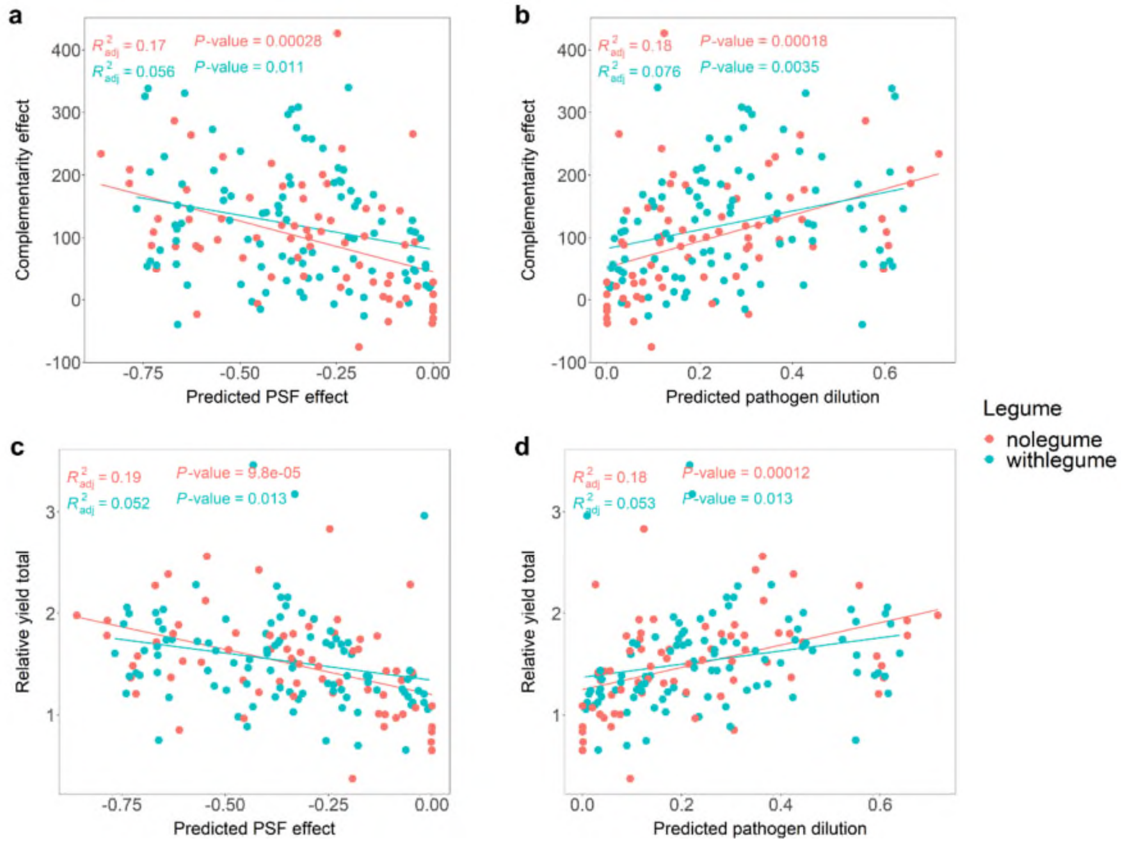

Supplementary Fig. 5 | The relationship between plant productivity and plant-soil feedback and pathogen dilution inclusion or exclusion of N-fixing legumes. a,b, Regressions between complementarity and predicted PSF effect (**a**) and pathogen dilution effect (**b**) inclusion (withlegume) or exclusion (nolegume) of N-fixing legumes. **c,d,** Regression between relative yield total and predicted PSF effect (**c**) and pathogen dilution effect (**d**) inclusion (withlegume) or exclusion (nolegume) of N-fixing legumes. Source data are provided as a Source Data file.

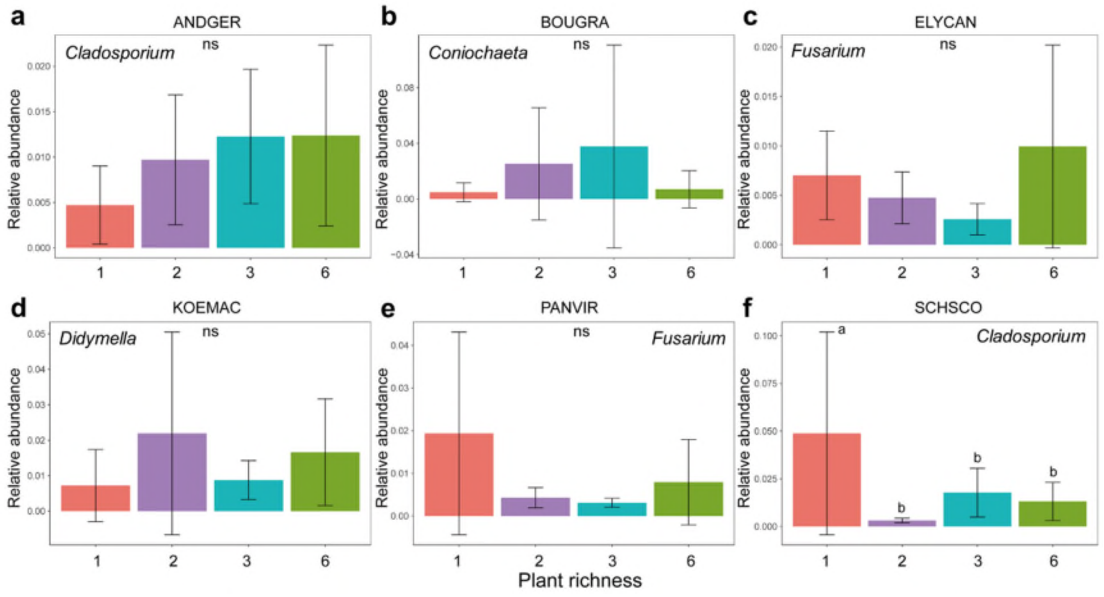

Supplementary Fig. 6 | The relative abundance changes of the most abundant pathogen species. a-f The relative abundance changes of the most abundant pathogen species at the genus level for each grass species, ANDGER (a), BOUGRA (b), ELYCAN (c), KOEMAC (d), PANVIR (e), SCHSCO (f). The full names of each species were provided at Supplementary Table 8. The most abundant genus was shown at the top left or top right corner of each plot. Bar plots indicate means ± standard error (SE). Source data are provided as a Source Data file.

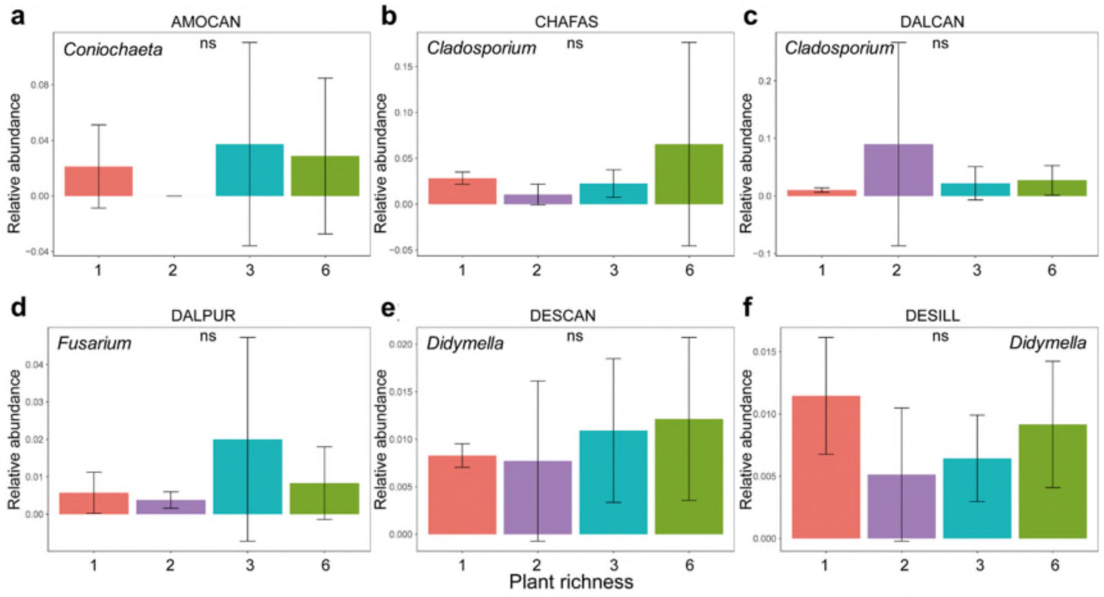

Supplementary Fig. 7 | The relative abundance changes of the most abundant pathogen species. a-f The relative abundance changes of the most abundant pathogen species at the genus level for each legume species, AMOCAN (a), CHAFAS (b), DALCAN (c), DALPUR (d), DESCAN (e), DESILL (f). The full names of each species were provided at Supplementary Table 8. The most abundant genus was shown at the top left or top right corner of each plot. Bar plots indicate means ± standard error (SE). Source data are provided as a Source Data file.

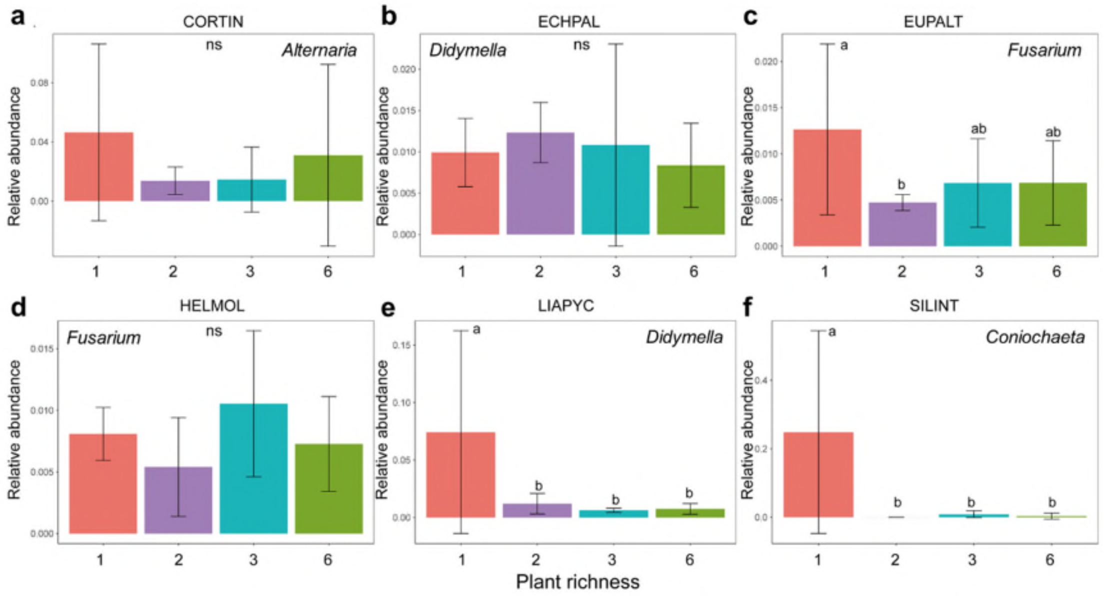

Supplementary Fig. 8 | The relative abundance changes of the most abundant pathogen species. a-f The relative abundance changes of the most abundant pathogen species at the genus level for each composite species, CORTIN (a), ECHPAL (b), EUPALT (c), HELMOL (d), LIAPYC (e), SILINT (f). The full names of each species were provided at Supplementary Table 8. The most abundant genus was shown at the top left or top right corner of each plot. Bar plots indicate means ± standard error (SE). Source data are provided as a Source Data file.

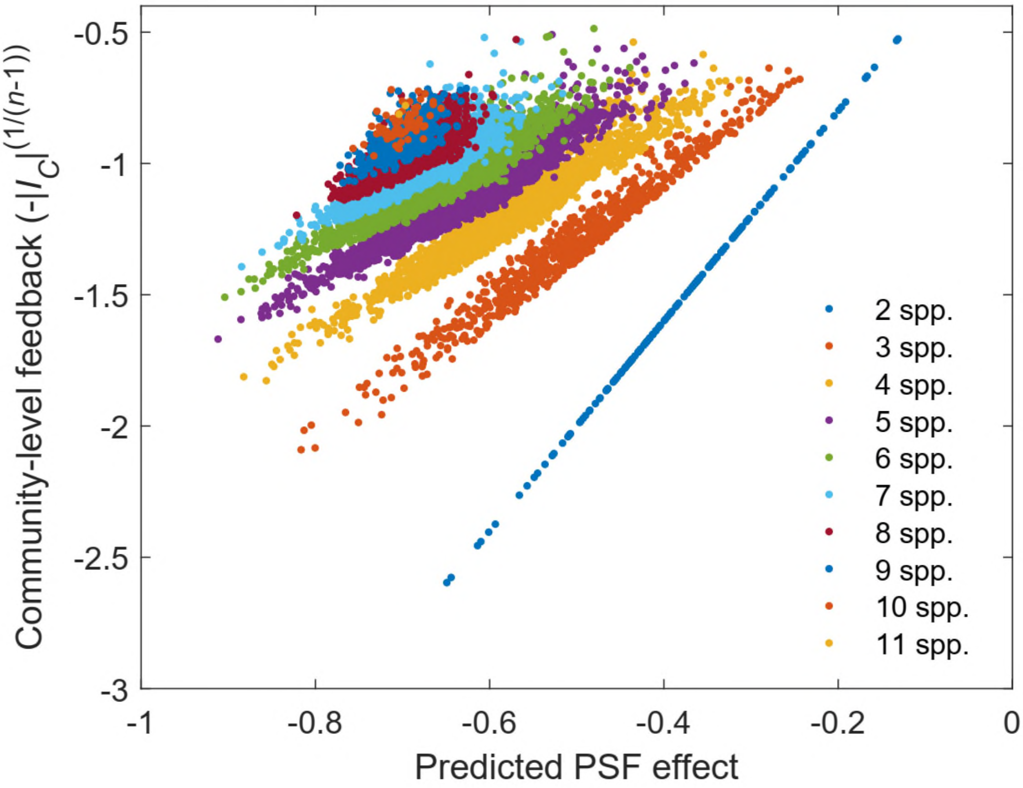

Supplementary Fig. 9 | Relationships between predicted PSF effect and community-level feedback. Model analyses of community-level feedback I_C was well predicted by the average plant-soil feedback that was constructed to analyze the empirical data. Negative community-level feedback, $I_C < 0$, is a necessary (but not sufficient) condition for coexistence of all plant species within a community. Source data are provided as a Source Data file.

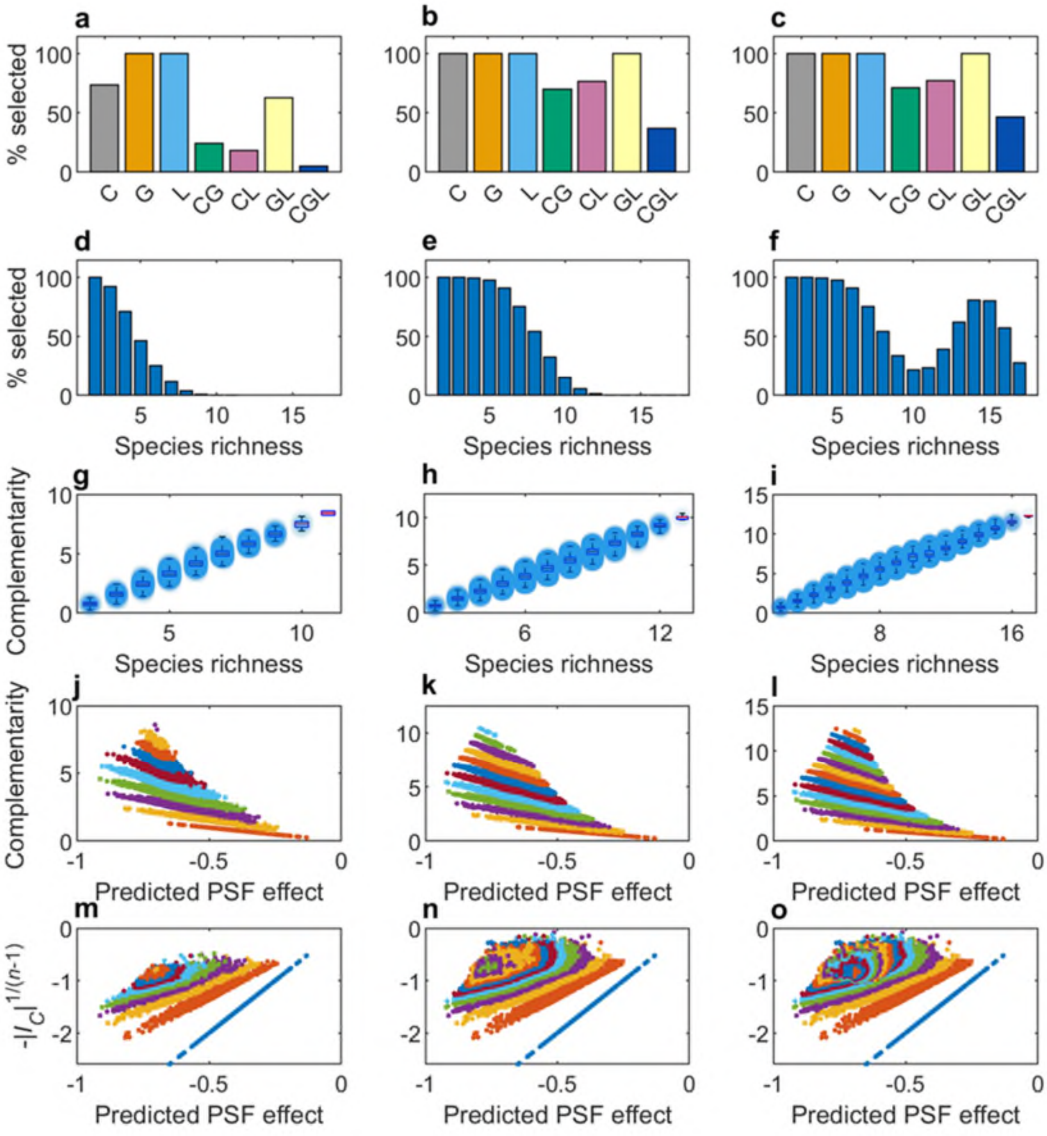

Supplementary Fig. 10 | Feedback-complementarity relationships under varying modelling approaches. The 18 species included in the empirical experiments allowed for model analysis of the 262,443 different plant communities that could be constructed from this species pool. In the main text, communities were selected if there was community-level negative feedback, and the coexistence equilibrium was feasible and locally stable (a, d, g, j, m). For less conservative approaches, relativizing the interaction matrix (b, e, h, k, n) and removing the local stability criterion (c, f, i, l, o) more diverse communities could be generated, up to all 18 species, but the main associations between species richness, predicted PSF effect and complementarity were similar across approaches. “C”, “G” and “L” represent composite, grass and legume respectively.

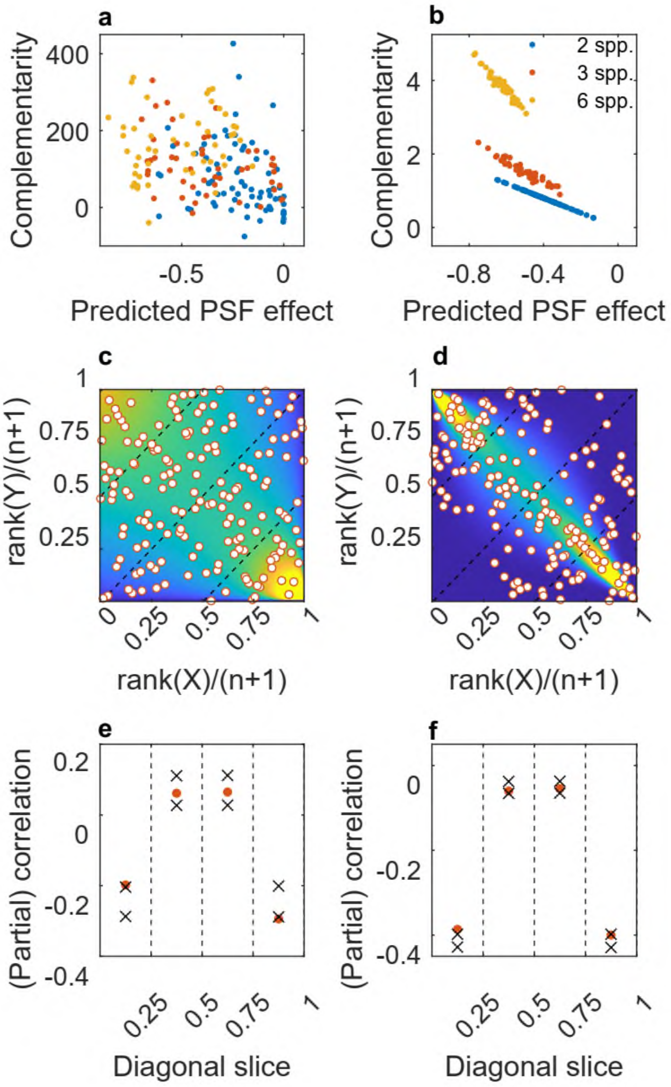

Supplementary Fig. 11 | Patterns of tail associations across the empirical and theoretical datasets. Statistical analysis of the copulas fitted to the empirical (a) and theoretical (b) datasets reveals asymmetric tail dependencies of the marginal distributions. Specifically, the best-fitting copulas on the empirical data (c) and theoretical data (d) suggested stronger upper-tail associations. This trend was also reflected by partial correlations within the empirical (e) and theoretical (f) data. The diagonal slices in panels e and f correspond to the regions demarcated by the black dashed lines in panels c and d.

		Composite						Grass						Legume					
		CORTIN	EUPALT	SILINT	LIAPYC	HELMOL	ECHPAL	ELYCAN	PANVIR	BOUGRA	KOEMAC	SCHSCO	ANDGER	CHAFAS	DESCAN	DESILL	DALPUR	DALCAN	AMOCAN
Composite	CORTIN																		
	EUPALT																		
	SILINT																		
	LIAPYC																		
	HELMOL																		
	ECHPAL																		
Grass	ELYCAN																		
	PANVIR																		
	BOUGRA																		
	KOEMAC																		
	SCHSCO																		
	ANDGER																		
Legume	CHAFAS																		
	DESCAN																		
	DESILL																		
	DALPUR																		
	DALCAN																		
	AMOCAN																		

Supplementary Fig. 12 | Composite, grass and legume species chosen for the plant-soil feedback test experiment (Experiment 2). The yellow, grey and light blue color represent plant species grown in their own soil, soil from the same family and from another two families, respectively. The full name of plant species were provide in Supplementary Table S8.

Supplementary Tables

Supplementary Table 1 | Results of the ANOVA test. Results of the ANOVA test for whether the PSFs with and between families were significantly differed from zero using the *rma.mv* function in *metafor* package, with the restricted maximum likelihood method.

Within and between family	Estimated value	SE (standard error)	***P***	95% CI	
				Lower	Upper
**Within composite**	-1.47	0.22	< **0.0001**	-1.90	-1.04
**Within grass**	-0.40	0.16	**0.0106**	-0.71	-0.09
**Within legume**	0.56	0.17	**0.001**	0.23	0.89
**Composite-grass**	-1.15	0.14	< **0.0001**	-1.42	-0.87
**Composite-legume**	-0.26	0.15	**0.079**	-0.55	0.03
Grass-legume	0.03	0.12	0.807	-0.20	0.26
**Average**	-0.36	0.061	< **0.0001**	-0.48	-0.24

Supplementary Table 2 | Regressions between microbial dissimilarity and pairwise PSF values. The statistical test used is F-test based on one-sided test, and significant effects ($P \leqslant 0.05$) denotes the overall significance of the regression model and are noted with bold font.

Microbial groups	Correlation coefficient	*P* value
**Soil oomycete**	**-0.249**	**0.025**
**Soil fungal pathogen**	**-0.219**	**0.050**
Soil AMF	-0.195	0.081
Root fungal pathogen	-0.190	0.089
Root fungal saprobe	-0.137	0.221
Soil non-rhizobia	-0.045	0.692
Root non-rhizobia	-0.044	0.695
Root AMF	-0.001	0.993
Root rhizobia	0.030	0.793
Soil fungal saprobe	0.086	0.446
Root oomycete	0.134	0.231

Supplementary Table 3 | Predicted model parameters for different microbial community groups. The statistical test used is F-test based on one-sided test, and significant effects ($P \leqslant 0.05$) denotes the significance of the model parameter and are noted with bold font.

	**Estimate**	**Adjusted Standard Error**	*P* value	**Weight**
(Intercept)	1.568	2.444	0.521	-
**Soil oomycete**	**-2.27**	**1.029**	**0.028**	**0.81**
**Soil fungal pathogen**	**-1.52**	**0.726**	**0.037**	**0.78**
Soil AMF	-1.66	1.182	0.162	0.47
**Root fungal pathogen**	**-1.27**	**0.648**	**0.049**	**0.71**
Root fungal saprobe	-0.97	0.944	0.304	0.35
Soil non-rhizobia	-0.53	2.669	0.843	0.23
Root non-rhizobia	-0.43	1.278	0.738	0.24
Root AMF	0.36	1.525	0.813	0.23
Root rhizobia	0.84	1.177	0.477	0.28
Soil fungal saprobe	1.51	0.869	0.082	0.62
Root oomycete	1.80	1.107	0.103	0.57

Supplementary Table 4 | Results of the t-test. Results of the two-sided t-test for testing whether the complementarity effect and relative yield totals in richness levels 2-6 were significantly greater than 0 and 1, respectively. Significant effects ($P \leqslant 0.05$) are noted with bold font.

**Plant richness**	**Complementarity effect**				**Relative yield total**			
	DF	t	Estimated means	*P*	DF	t	Estimated means	*P*
2	71	7.14	76.86	< **0.001**	71	5.95	1.36	< **0.001**
3	35	9.98	111.22	< **0.001**	35	8.84	1.55	< **0.001**
6	35	13.49	165.66	< **0.001**	35	15.09	1.71	< **0.001**

Supplementary Table 5 | Results of the ANOVA test on plant productivity and biodiversity effects. Results of the ANOVA test for the effects of phylogenetic dispersion (within family, between family), plant richness (2, 3, 6) and their synergistic effects on plant biomass, complementarity effect and relative yield total of mixture plots. The statistical test used is F-test based on one-sided test, and significant effects ($P \leqslant 0.05$) denotes the significance of the model parameter and are noted with bold font.

	**Plant biomass**			**Complementarity effect**			**Relative yield total**		
	DF	F	*P*	DF	F	*P*	DF	F	*P*
Dispersion	1	1.167	0.282	1	0.212	0.646	1	0.236	0.627
Plant richness	2	14.61	**< 0.001**	2	15.22	**< 0.001**	2	9.072	**< 0.001**
Dispersion*Plant richness	2	0.043	0.958	2	0.199	0.82	2	0.682	0.507
Residuals	162			162			162		

Supplementary Table 6 | Results of the ANOVA test. Results of the ANOVA test for the effects of phylogenetic dispersion (within family, between family) on plant pairwise feedback, soil fungal pathogen community dissimilarity and soil oomycete community dissimilarity. The statistical test used is F-test based on one-sided test.

	**Pairwise feedback**			**Soil fungal pathogen dissimilarity**			**Soil oomycete dissimilarity**			**Root fungal dissimilarity**		
	DF	F	*P*	DF	F	*P*	DF	F	*P*	DF	F	*P*
Dispersion	1	0.215	0.644	1	0.606	0.437	1	0.120	0.730	1	0.159	0.69
Residuals	79			151			151			151		

Supplementary Table 7 | Results of the ANOVA test. Significant difference tests on the regression models between complementarity effect and relative yield total and predicted PSF and pathogen dilution effect under withlegume (mixture plots with legumes) and nolegume (mixture plots without legumes) treatments. The statistical test used is F-test based on one-sided test, and non-significant effects (P > 0.05) are noted with bold font.

	**Complementarity effect**				**Relative yield total**			
	Estimate	SE (standard error)	t value	*P*	Estimate	SE (standard error)	t value	*P*
Intercept	45.46	17.97	2.53	0.0123	1.20	0.09	13.21	< 0.001
Predicted PSF	-162.51	43.96	-3.70	<0.001	-0.89	0.22	-4.00	< 0.001
Legume	35.96	24.99	1.44	0.15	0.14	0.13	1.12	0.26
Predicted PSF × Legume	53.68	60.1	0.89	**0.37**	0.35	0.30	1.16	**0.25**
Intercept	52.69	16.01	3.29	0.0012	1.25	0.08	15.28	< 0.001
Predicted pathogen dilution (PPD)	208.73	54.46	3.83	<0.001	1.10	0.28	3.94	< 0.001
Legume	30.12	22.14	1.36	0.18	0.12	0.11	1.08	0.28
PPD × Legume	-59.17	73.17	-0.81	**0.42**	-0.45	0.37	-1.20	**0.23**

Supplementary Table 8 | All 18 species used in this study.

Family	Name	Abbreviation
Asteraceae	*Coreopsis tinctoria*	CORTIN
	Echinacea pallida	ECHPAL
	Eupatorium altissimum	EUPALT
	Helianthus mollis	HELMOL
	Liatris pycnostachya	LIAPYC
	Silphium integrifolium	SILINT
Poaceae	*Andropogen gerardii*	ANDGER
	Bouteloua gracilis	BOUGRA
	Elymus canadensis	ELYCAN
	Koeleria macrantha	KOEMAC
	Panicum virgatum	PANVIR
	Schizachyrium scoparium	SCHSCO
Fabaceae	*Amorpha canescens*	AMOCAN
	Chamaecrista fasciculata	CHAFAS
	Dalea candida	DALCAN
	Dalea purpurea	DALPUR
	Desmodium canadense	DESCAN
	Desmodium illinoense	DESILL

Supplementary Discussion

Relationships between biotic effects and plant diversity benefits with and without legumes

To account for the underlying effect of legumes, plants which can form symbioses with N-fixing rhizobia, and have documented impacts on plant productivity[1,2], we conducted further analyses to determine whether the inclusion or exclusion of legumes influenced the relationship between predicted PSF and complementarity or RYT. Similar trends were found for 'withlegume' (mixture plots with legumes) and 'nolegume' (mixture plots without legumes) groups (Supplementary Fig. 5). Specifically, the interaction between predicted PSF or pathogen dilution in communities including or without legumes were not significant, suggesting the effects of soil microbiomes on overyielding were not significantly altered by legumes (Supplementary Table 7). These results indicate that biotic PSF effects can explain the plant biodiversity effects, even in plant mixtures with legumes.

Relative abundance changes of plant pathogen in different plant richness

To evaluate the role of specialist pathogens in generating PSF effects, we calculated the relative abundance of the fungal pathogens for each plant species based on the amplicon sequencing data. We note that this is an imperfect measure as it is possible that the overall density of pathogenic fungi is changing across plots (e.g. it could decline with diversity), which would not be detected with amplicon sequencing. However, currently it is methodologically and quantitatively infeasible to scan for specific pathogens across 18 plant species, as we have in our study. Nevertheless, using this approach, we found that the relative abundance of specific pathogens declined with diversity in four examples (Supplementary Figs. 6-8). *Cladosporium* in SCHSCO (Supplementary Fig. 6f), *Fusarium* in EUPALT (Supplementary Fig. 8c), *Didymella* in LIAPYC (Supplementary Fig. 8e) and *Coniochaeta* in SILINT (Supplementary Fig. 8f) were significantly reduced in monoculture compared with mixtures, in part supporting the pathogen dilution hypothesis.

PSF-complementarity relationships within and across species richness levels

We found that predicted PSF effects strongly correlated with community-level feedback (Supplementary Fig. 9). The strength of the correlation slightly decreases as the number of species in the communities increases, which can be partly attributed to the importance and number of higher order interactions increasing with community size. Moreover, the absolute value of I_C decreases with community size according to the exponent $1/(N\text{-}1)$, which means that relatively small differences in I_C are increasingly amplified with larger community sizes (increasing the variance within the variable). As the correlation between pairwise feedback and community-level feedback depends on the particular structure of the interaction matrix, these results suggest that the empirical constraints on the interaction matrix yield an interaction structure under which pairwise interactions are an important component of community-level feedback[3].

Robustness of feedback-complementarity relationships under varying modelling approaches

We found that under the less conservative approaches, a larger proportion of the assembled communities showed the potential to coexist, with the largest increases occurring for communities including more than 10 species from multiple families (Supplementary Fig. 10a-f). For example, 5 (out of 18) of the 17-species communities exhibited negative community-level feedback. We found, however, that the linear increase in complementarity with species richness emerged in all three modelling approaches (Supplementary Fig. 10g-i). Moreover, we found that complementarity increased with the strength of negative feedback, and that the slope of the relationship increased with species richness (Supplementary Fig. 10j-l). As more diverse communities were also characterized by more strongly negative feedback the asymmetric upper-tail dependency of this relationship was also observed for all three approaches. Hence, we can conclude that the findings presented in the main text were

robust to the specific modelling approach utilized. With regard to the correlation between predicted PSF effect and community-level feedback, we saw that the strength of this correlation continued to weaken when the more diverse communities within the second and third modelling approaches considered (Supplementary Fig. 10m-o). As noted in the preceding section, this trend can at least in part be explained by the increase in species richness, which increases the variance in rescaled community-level feedback and increases the number of higher order interactions in the system.

Patterns of tail associations across the empirical and theoretical datasets

The empirical data (Figure 4a of the main text) was best described by a Clayton cupola that indicated stronger association between predicted PSF and complementarity at the upper tails of the marginal distributions (Clayton cupola: $(u^{-0.62}+ v^{-0.62}-1)^{1/0.62}$, Information matrix test statistic: 0.04, p = 0.80; Lower-tail dependence: 0, Upper-tail dependence: 0.33). Indeed, this asymmetry in tail associations was reflected in the correlation coefficient being significantly lower at the lower tail, and significantly higher at the upper tail than expected from a symmetric distribution (Spearman correlation coefficients: $\rho_{lower} = 0.20$, p = 0.02; $\rho_{upper} = 0.29$, p = 0.03) (Supplementary Fig. 11a,c,e). This association pattern suggests that complementarity declined relatively strongly in plots exhibiting weaker PSF effects. For the simulated data, we randomly selected 100 subsets of the 17,850 2-, 3-, and 6-species communities that exhibited negative community-level feedback. Matching the empirical dataset, we included 72 2-species communities and 48 3-species and 6-species communities in each subset (see Methods description in the main text). For these subsets, the Joe-Clayton copula was most often selected as the best descriptor of the data (71 of the 100 subsets). Similar to the empirical data fit, a typical fit of this copula to a randomly drawn subset of the simulated data would also indicate a stronger association between predicted PSF and complementarity at the upper tails of the marginal distributions (e.g., Joe-Clayton copula: $1-(1-(-1+(1-(1-u)^{3.13})^{-6}+(1-(1-v)^{3.13})^{-6})^{-1/6})^{1/3.13}$; Cramer-von Mises test statistic: 0.06, p = 0.17; Lower-tail dependence: 0.75, Upper-tail dependence: 0.89). This

asymmetry in tail associations was reflected in the correlation coefficient at the lower tail being significantly lower than expected from a symmetric distribution (Supplementary Fig. 11b,d,f), Spearman correlation coefficients: $\rho_{lower} = 0.39$, $p = 0.007$; $\rho_{upper} = 0.40$, $p = 0.08$). Again, this association pattern is consistent with complementarity declining relatively strongly under weaker PSF effects.

As noted in the main text, the asymmetric tail associations described above could be partly explained by the stepwise progression across species richness levels of higher levels of complementarity and predicted PSF effects. Specifically, in the empirical data, we only found disproportionate upper-tail dependence of the association for 2-species communities (2-species communities: Clayton cupola: max $(u^{-0.62} + v^{-0.62} - 1, 0)$, Information matrix test statistic: 0.10, $p = 0.58$; Lower-tail dependence: 0, Upper-tail dependence: 0.33). In contrast, no evidence for asymmetric tails associations was found for 3-species communities (Gaussian copula) or 6-species communities (Independence copula). Similarly, the most frequently selected copulas for randomly selected subsets of simulated 2- (*t* copula) 3-species (Gaussian copula) and 6-species communities (Gaussian copula) did not exhibit asymmetric tail associations. Hence, these results suggest that the upper-tail dependence of the association between predicted PSF effects and complementarity is driven by the combination of species richness impacts on complementarity and average PSF of coexisting communities, and the dependence of complementarity on average PSF within species richness levels (Supplementary Fig. 10).

Supplementary Methods

Seeds preparation and planting in Experiment 1

Prairie seeds were purchased from producers located near eastern Kansas (Hamilton Native Outpost, Stock Seed, Missouri Wildflowers, and Prairie Moon) and planted in May 2018. Each plot was seeded with 1800 seeds divided equally amongst the species in the plot. Resident soil microbes were augmented with soil microbes from native prairie in two ways. Firstly, we added 4 cm of soil from an unplowed native prairie soil from Welda, KS, which was then tilled into the resident soil to a depth of 15 cm. Secondly, as native microbes may not be resilient to tillage, we also introduced microbes by planting nurse plants. Briefly, seeds were sowed into flats with autoclaved sterile potting soil and placed in cold-moist stratification for four weeks prior to germination. When large enough, the seedlings were transplanted into groove tubes (GT51D, Stuewe and Sons, Oregon) with inoculation of 98 mL of fresh soil from unplowed prairie remnant from Welda, KS and grown in a greenhouse for 5 weeks prior to being planted. Nurse plants were inoculated with native soil microbes and then grown for two months prior to planting in the field to ensure that the microbes had a chance to establish on their host plants. For each plot, 18 seedlings previously inoculated with native soil were planted into a hexagonal array. These 18 seedlings were divided between the species planted into the plot, with different species being equally spaced.

The plots were watered every other day for two weeks following planting to facilitate establishment and thereafter received ambient precipitation. Plots were weeded starting one month after planting with care taken not to damage planted or seeded seedlings. Starting in August 2018, the high water treatment, selected from the paired shelters at random, received supplemental irrigation to 150% ambient growing season precipitation (April 1st-October 31st) totaling 654.8 and 415.9 mm for high and low treatments respectively. During the growing season, this site receives on average 762.3 mm precipitation. Since the summer of 2018 was already a drought year and the rainfall exclusion shelters were being constructed, the precipitation treatment was not fully realized until the second year of the experiment. Given that our soils were sampled

in September with little time for precipitation treatments to manifest, we pooled soil samples from paired plots across the precipitation treatment for analysis of microbiome composition and greenhouse tests of plant-soil feedbacks. Seeds were reapplied as above in the February 2019. During the winter (Nov 1st-March 31st) the plots received ambient precipitation at which point films were installed on the shelters and precipitation treatments were fully realized. Plots were weeded from mid-May to mid-June 2019, just after plant emergence for the planted species (last week April 2019) and finished one month prior to biomass harvest (July 2019).

Theoretical analysis

In the main text, it was noted that our simulations demonstrate that the negative feedbacks observed in this study can stabilize communities. This is evident in the communities represented in Figure 5 and the supplementary figures, which suggest that communities would be coexisting at equilibrium because of feedbacks parameterized from observed pairwise pathogen dissimilarities. We also illustrate this point by noting that the approach to estimate predicted PSF effects (eq. 4) yielded a metric that was strongly correlated with community-level feedback, the metric that determines stability of multispecies communities structured by feedback[3]. The community level feedback is defined by Eppinga, et al. [3] as:

$$I_C = (-1)^N \sum_{j=1}^{N} \det \mathbf{A}_j \qquad \text{(S1)}$$

In which $\mathbf{A}$ is the community interaction matrix (see equation 11 in the Methods section), with the subscript j indicating that the coefficients of the jth column have been replaced by a column vector of ones of length N, where N indicates the number of plant species within the community. It has been proven mathematically that negative community-level feedback is a necessary (but not sufficient) condition for community coexistence[3]. For the theoretical communities as obtained by parameterizing the interaction matrix with the empirical estimates of pair-wise feedbacks, we calculated the predicted PSF effects (see equation 4 in the Methods), using the equilibrium frequencies[3]:

$$p_i = \frac{\det \mathbf{A}_i}{I_C} \qquad \text{(S2)}$$

In the Methods section of the main text, it was noted that analytical model analysis of assembled communities was restricted to communities for which the interaction matrix suggested that there was negative community-level feedback, the coexistence equilibrium was feasible and locally stable. This approach assesses the potential of feedbacks to drive community coexistence conservatively, as local stability is not a necessary requirement for species persistence[3,4]. Moreover, biomass responses may require adjustment to serve as a measure of relative fitness of phylogenetically dissimilar plant species[5,6]. Here, we compared the approach presented in the main text with two alternative modelling approaches to estimate the interaction matrix, to test the robustness of the findings presented in the main text. For these alternative approaches, we followed the procedure described in a previous study to relativize measured plant-soil community interactions among different plant species[5]. This relativization procedure involves subtracting from each coefficient in the interaction matrix the row mean, and add the overall interaction matrix mean. Subsequently, we either included or omitted the requirement of the real part of the dominant eigenvalue being negative. Hence, the latter approach focuses solely on negative community-level feedback as a requirement, and is therefore the least conservative of the three approaches.

It was noted that the relationship between predicted PSF effects and complementarity is composed of two components: a positive relationship within plot species richness level, and a stair step progression across species richness levels of higher average complementarity and more negative average feedback. We examined this relationship in more detail through the construction of copulas, which allow for a mathematically complete description of the association between two variables[7-9]. Here, we were particularly interested in evaluating the extent to which associations between predicted PSF effects and complementarity were characterized by stronger or weaker association in the tails of the marginal distributions. Specifically, we aimed to test whether these patterns of tail associations were consistent across the empirical and theoretical datasets.

For each dataset, the best-fitting copula was selected from a family of 1-parameter and 2-parameter copulas and the goodness-of-fit evaluated using the *BiCopSelect* and *BiCopGofTest* functions as implemented in the *VineCopula* R package[10]. As a satisfactory fit was obtained for all datasets (i.e. no rejection of the null hypothesis at the 0.05 probability level that the copula and the data are similar), the degree of tail dependence was then obtained through the *BiCopPar2TailDep* function implemented in the same package. As the degree of tail dependence is not straightforwardly utilized in hypothesis testing, we followed the approach described in Ghosh, et al. [9], which tests the strength of tail dependence in the relationship (between variables *x* and *y*) through a non-parametric correlation and bootstrapping approach applied to segments of the rank-transformed data (here referred to as variables *u* and *v*. Bootstrap replicates were generated using the *copsurrog2d* function as implemented in the *BIVAN* R package[9].

Supplementary References

1. Fargione, J. et al. From selection to complementarity: shifts in the causes of biodiversity–productivity relationships in a long-term biodiversity experiment. *Proc. Royal Soc. B* **274**, 871-876 (2007).
2. Marquard, E. et al. Plant species richness and functional composition drive overyielding in a six-year grassland experiment. *Ecology* **90**, 3290-3302 (2009).
3. Eppinga, M. B. et al. Frequency-dependent feedback constrains plant community coexistence. *Nat. Ecol. & Evol.* **2**, 1403-1407 (2018).
4. Revilla, T. A. et al. Plant-soil feedbacks and the coexistence of competing plants. *Theor. Ecol.* **6**, 99-113 (2013).
5. Mangan, S. A. et al. Negative plant-soil feedback predicts tree-species relative abundance in a tropical forest. *Nature* **466**, 752-755 (2010).
6. Mack, K. M., Eppinga, M. B. & Bever, J. D. Plant-soil feedbacks promote coexistence and resilience in multi-species communities. *PLoS One* **14**, e0211572 (2019).
7. Joe, H. *Dependence modeling with copulas* (CRC press, 2014).
8. Mai, J.-F. & Scherer, M. *Simulating copulas: stochastic models, sampling algorithms, and application*s (World Scientific Publishing Company, 2017).
9. Ghosh, S. et al. Copulas and their potential for ecology. *Adv. Ecol. Res.* **62**, 409-468 (2020).
10. Nagler, T. et al. Package ‘VineCopula’. Available at: https://CRAN.R-project.org/package=VineCopula (2022).
