# Supplementary material for: Dilution of specialist pathogens drives productivity benefits from diversity in plant mixtures
Source: Nat Commun. 2023 Dec 18;14:8417. doi: 10.1038/s41467-023-44253-4 (PMC10728191; doi:10.1038/s41467-023-44253-4)
Supplement: Supplementary file 3 — Reporting Summary [file 41467_2023_44253_MOESM3_ESM.pdf]

nature portfolio

Corresponding author(s): Guangzhou Wang, James Bever

Last updated by author(s): Nov 30, 2023

Reporting Summary

Nature Portfolio wishes to improve the reproducibility of the work that we publish. This form provides structure for consistency and transparency in reporting. For further information on Nature Portfolio policies, see our Editorial Policies and the Editorial Policy Checklist.

Statistics

For all statistical analyses, confirm that the following items are present in the figure legend, table legend, main text, or Methods section.

n/a	Confirmed	
☐	☒	The exact sample size (*n*) for each experimental group/condition, given as a discrete number and unit of measurement
☐	☒	A statement on whether measurements were taken from distinct samples or whether the same sample was measured repeatedly
☐	☒	The statistical test(s) used AND whether they are one- or two-sided<br>*Only common tests should be described solely by name; describe more complex techniques in the Methods section.*
☐	☒	A description of all covariates tested
☐	☒	A description of any assumptions or corrections, such as tests of normality and adjustment for multiple comparisons
☐	☒	A full description of the statistical parameters including central tendency (e.g. means) or other basic estimates (e.g. regression coefficient) AND variation (e.g. standard deviation) or associated estimates of uncertainty (e.g. confidence intervals)
☐	☒	For null hypothesis testing, the test statistic (e.g. *F*, *t*, *r*) with confidence intervals, effect sizes, degrees of freedom and *P* value noted<br>*Give P values as exact values whenever suitable.*
☒	☐	For Bayesian analysis, information on the choice of priors and Markov chain Monte Carlo settings
☒	☐	For hierarchical and complex designs, identification of the appropriate level for tests and full reporting of outcomes
☐	☒	Estimates of effect sizes (e.g. Cohen's *d*, Pearson's *r*), indicating how they were calculated

Our web collection on statistics for biologists contains articles on many of the points above.

Software and code

Policy information about availability of computer code

Data collection	No software was used for data collection.
Data analysis	QIIME2 (2019.10), SILVA, UNITE(V 18.11.2018), FungalTraits, BLAST (v. 2.6.0), vegan package(v 3.5.1), metafor package, ggpmisc package, A3 package, glmulti package. All codes used in this study are available on GitHub (https://github.com/wlzwgz/Dimension-PSF.git).

For manuscripts utilizing custom algorithms or software that are central to the research but not yet described in published literature, software must be made available to editors and reviewers. We strongly encourage code deposition in a community repository (e.g. GitHub). See the Nature Portfolio guidelines for submitting code & software for further information.

Data

Policy information about availability of data

All manuscripts must include a data availability statement. This statement should provide the following information, where applicable:

- Accession codes, unique identifiers, or web links for publicly available datasets
- A description of any restrictions on data availability
- For clinical datasets or third party data, please ensure that the statement adheres to our policy

All raw data required to reproduce the results are provided in the Figshare (https://doi.org/10.6084/m9.figshare.23804619). Sequences were submitted to the NCBI Sequence Read Archive (SRA) under the accession number PRJNA863284.

Research involving human participants, their data, or biological material

Policy information about studies with human participants or human data. See also policy information about sex, gender (identity/presentation), and sexual orientation and race, ethnicity and racism.

Reporting on sex and gender	NA
Reporting on race, ethnicity, or other socially relevant groupings	NA
Population characteristics	NA
Recruitment	NA
Ethics oversight	NA

Note that full information on the approval of the study protocol must also be provided in the manuscript.

Field-specific reporting

Please select the one below that is the best fit for your research. If you are not sure, read the appropriate sections before making your selection.

☐ Life sciences ☐ Behavioural & social sciences ☒ Ecological, evolutionary & environmental sciences

For a reference copy of the document with all sections, see nature.com/documents/nr-reporting-summary-flat.pdf

Ecological, evolutionary & environmental sciences study design

All studies must disclose on these points even when the disclosure is negative.

Study description	We tested whether pathogen dilution generates diversity-productivity relationships using a field biodiversity-manipulation experiment (altering plant diversity and composition), greenhouse assays, and feedback modeling. For the field experiment, We manipulated species richness (1, 2, 3, and 6), plant community composition (phylogenetically under or over dispersed), and precipitation (50 or 150% ambient rainfall). The full-factorial design comprised 72 monocultures, 72-two species, 48-three species, and 48-six species plots, with each of the 18 plant species being equally represented in all treatment combinations. For the greenhouse experiment, each of the 18 species were grown with inocula from three plant species monoculture plots from each family, which resulted in 81 full factorial pairwise feedback tests. For each of 18 species, one individual of each species was planted, with their own monoculture soils (nine replicates) and nine soil treatments for other species (three replicates of three heterospecific species from each family), and sterile soil was used for controls, including three replicates for each species.
Research sample	In the field plant diversity manipulation experiment, a total of 240 plots (1.5 m × 1.5 m) were established, with 18 native prairie plant species chosen from three families. Bacterial, fungal, oomycete, and AM fungal communities were sequenced from both soil and root DNA samples (960 samples in total). Then we conducted the PSF feedback experiment using all 18 species and grew them with pots inoculated with field soils from conspecific monocultures, or monocultures from other species from the same family, or from a different family, resulting in 702 pots in total. The complementarity effect and relative yield total of all 168 plant mixture plots of next year were calculated for assessing their relationships with biotic PSFs. The general feedback model simulated the expected relationships between complementarity, species richness, predicted plant-soil feedback (PSF) effects and predicted pathogen dilution when plant community dynamics are driven by host-specific soil pathogens, with a theoretical data number of 17, 850.
Sampling strategy	Soils were collected from each plot and then paired plots of matched plant composition were pooled across paired rainfall exclusion shelters. A total of six 20 cm soil cores with 1.9 cm diameter (approximately 340 ml) were taken from each plot. The coring devices were sanitized between different treatments to minimize contamination. The soil cores were taken close to planted species to be more representative of plant-affected soil microbes and to ensure we were able to collect root samples. Plant aboveground biomass was harvested at the peak biomass, weighed from 0.1m2 strips and plant cover surveys were conducted for the total plot. Our sampling in total have 120 soil samples and 120 root samples, and 240 plot level biomass data. The statistical analyses were based on the collected soil and plant samples and the processing methods were described in the Methods sections.
Data collection	DNA were extracted from 0.25 g fresh soil or root samples following the manufacturer's instructions (DNeasy PowerSoil Kit, Qiagen, Hilden, Germany). PCR products were barcoded using Nextera XT Index Kit v2 (Illumina, San Diego, CA, USA) for indexing and purified using AMPure XP beads (Beckman Coulter, Brea, CA, USA). PCR products concentration was measured by Invitrogen Qubit 3.0 Fluorometer (Thermo Fisher Scientific, Waltham, MA, USA). Sequencing was performed by Illumina MiSeq v3 PE300 Next-Gen Sequencer in Genome Sequencing Core of University of Kansas. Both aboveground and root biomass were harvested for the greenhouse experiment, and aboveground plant biomass of field experiment was sorted to species within one day, dried at 70°C for at least three days, and weighed. G.W., H.M.B., L.Y. P. and P.S. performed the field work, and G.W. performed the greenhouse experiments. G.W. and H.M.B. did DNA extraction and sequencing processing work.

Timing and spatial scale	The field experiment was established in May 2018 and the soil samples were collected at September 2018. The greenhouse experiment was conducted between May and July 2019. The field plant biomass was harvested In July 2019. Soil samples were taken by cores with 1.9 cm diameter (approximately 340 ml). The greenhouse experiment was conducted using the deep-pot (diameter 6.4cm, height 25.4 cm) filled with 500 mL soil. Plant aboveground biomass in the field was harvested and weighed from 0.1m2 strips and plant cover surveys were conducted for the total plot.
Data exclusions	All data was used.
Reproducibility	For all the analyses, we have at least 9 replicates. In particular, with hundreds of plots, we are able to replicate monocultures of all planted species, and we are able to equally represent each planted species in each treatment combination.
Randomization	We used the randomized block design for both our field and greenhouse experiments, and when doing the statistics, the random effect was considered.
Blinding	The process of data collection and analysis is blind before relevant results are revealed.

Did the study involve field work? ☒ Yes ☐ No

Field work, collection and transport

Field conditions	The field experiment was established in May 2018 at the University of Kansas Field Station (39°03'09" N, 95°11'30" W), located in eastern Kansas, USA. It has a continental climate characterized by warm, wet summers and cold, dry winters. The mean annual temperature is 12.7°C (55°F). The annual average precipitation is 990 mm and approximately 70% of it falls between April and September.
Location	The field experiment was established at the University of Kansas Field Station (39°03'09" N, 95°11'30" W)
Access & import/export	Soil and plant samples were collected in compliance with the local and national laws, and are authorized by the University of Kansas Field Station.
Disturbance	No disturbance was caused.

Reporting for specific materials, systems and methods

We require information from authors about some types of materials, experimental systems and methods used in many studies. Here, indicate whether each material, system or method listed is relevant to your study. If you are not sure if a list item applies to your research, read the appropriate section before selecting a response.

Materials & experimental systems

n/a	Involved in the study	
☒	☐	Antibodies
☒	☐	Eukaryotic cell lines
☒	☐	Palaeontology and archaeology
☒	☐	Animals and other organisms
☒	☐	Clinical data
☒	☐	Dual use research of concern
☐	☒	Plants

Methods

n/a	Involved in the study	
☒	☐	ChIP-seq
☒	☐	Flow cytometry
☒	☐	MRI-based neuroimaging

Dual use research of concern

Policy information about dual use research of concern

Hazards

Could the accidental, deliberate or reckless misuse of agents or technologies generated in the work, or the application of information presented in the manuscript, pose a threat to:

No	Yes	
☒	☐	Public health
☒	☐	National security
☒	☐	Crops and/or livestock
☒	☐	Ecosystems
☒	☐	Any other significant area

Experiments of concern

Does the work involve any of these experiments of concern:

No	Yes	
☒	☐	Demonstrate how to render a vaccine ineffective
☒	☐	Confer resistance to therapeutically useful antibiotics or antiviral agents
☒	☐	Enhance the virulence of a pathogen or render a nonpathogen virulent
☒	☐	Increase transmissibility of a pathogen
☒	☐	Alter the host range of a pathogen
☒	☐	Enable evasion of diagnostic/detection modalities
☐	☐	Enable the weaponization of a biological agent or toxin
☒	☐	Any other potentially harmful combination of experiments and agents

Plants

Seed stocks	Seeds were pur- chased from producers located near eastern Kansas: Hamilton Native Outpost, Stock Seed, Missouri Wildflowers, and Prairie Moon.
Novel plant genotypes	NA
Authentication	NA
